# Yellow Tongue Coating is Associated With Diabetes Mellitus Among Japanese Non-smoking Men and Women: The Toon Health Study

**DOI:** 10.2188/jea.JE20160169

**Published:** 2018-06-05

**Authors:** Kiyohide Tomooka, Isao Saito, Shinya Furukawa, Koutatsu Maruyama, Eri Eguchi, Hiroyasu Iso, Takeshi Tanigawa

**Affiliations:** 1 Department of Public Health, Juntendo University Graduate School of Medicine, Tokyo, Japan; 2 Department of Community Health Systems Nursing, Ehime University Graduate School of Medicine, Ehime, Japan; 3 Department of Epidemiology and Preventive Medicine, Ehime University Graduate School of Medicine, Ehime, Japan; 4 Department of Public Health, Okayama University Graduate School of Medicine, Dentistry and Pharmaceutical Sciences, Okayama, Japan; 5 Public Health, Department of Social Medicine, Osaka University Graduate School of Medicine, Osaka, Japan

**Keywords:** yellow tongue coating, diabetes mellitus, oral glucose tolerance test, traditional East Asian medicine, cross-sectional study

## Abstract

**Background:**

Yellow tongue coating is one of the clinical signs for diabetes mellitus according to traditional East Asian medicine. Few reports have been available on the association between yellow tongue coating and the prevalence of type 2 diabetes in the general population. We examined that association among population samples of non-smoking men and women.

**Methods:**

The study subjects were Japanese non-smoking men (*n* = 315) and women (*n* = 654) aged 30–79 years who resided in Toon city and participated in the Toon Health Study from July 2011 through November 2014. Tongue coating was assessed by a nationally licensed acupuncturist and classified into three categories of white (normal), light yellow, and yellow. We performed an oral glucose tolerance test to confirm the presence of diabetes mellitus and prediabetes. The associations between yellow tongue coating and the prevalence of diabetes mellitus and prediabetes were examined using multivariable logistic regression analyses, adjusting for age, sex, body mass index, drinking status, and physical activity.

**Results:**

The multivariable odds ratios of diabetes mellitus were 1.39 (95% confidence interval [CI], 0.72–2.67) for light yellow tongue coating and 2.23 (95% CI, 1.16–4.30) for yellow tongue coating compared with white tongue coating. The respective multivariable odds ratios of prediabetes were 1.13 (95% CI, 0.80–1.61) and 1.43 (95% CI, 0.96–2.12).

**Conclusions:**

Yellow tongue coating was associated with higher prevalence of diabetes mellitus and tended to be associated with that of prediabetes among Japanese non-smoking men and women.

## INTRODUCTION

Type 2 diabetes mellitus is one of the major lifestyle-related diseases around the world. According to the International Diabetes Federation estimates, 366 million people worldwide suffered from type 2 diabetes in 2011, and it is expected to affect a staggering 552 million people by the year 2030.^1^

Recently, complementary and alternative medicine, including traditional East Asian medicine, has been widely used for the treatment of type 2 diabetes mellitus and its complications around the world.^2^^–^^9^ However, the evidence for traditional East Asian medicine for the diagnosis, prevention, and treatment of type 2 diabetes is limited. Therefore, epidemiological studies are needed to assess the usefulness of traditional East Asian medicine for them.

Yellow discoloration of tongue coating is one of the classical clinical signs used for the assessment of diabetes mellitus in traditional East Asian medicine.^10^ Several clinical reports suggested that a yellow tongue coating is one of the concomitant symptoms of diabetes.^11^^,^^12^ The discoloration of tongue coating is associated with smoking, poor oral hygiene, coffee consumption, *Candida albicans* infection, periodontal disease, and the effect of medications, such as antibacterial and psychotropic agents.^13^^–^^17^ These factors are also associated with type 2 diabetes. Additionally, previous studies showed that tongue lesions were more prevalent among patient with type 2 diabetes than healthy controls,^18^^,^^19^ and hyperglycemia was a risk factor for the occurrence of tongue lesions.^20^ However, the evidence on the association between yellow tongue coating and type 2 diabetes has come from case-series studies in clinical settings^11^^,^^12^; no population-based observational study has been conducted for apparently healthy persons. Since smoking causes the discoloration of teeth, restorations, and dentures,^21^^,^^22^ and is also associated with the formation of tongue coating,^23^ we restricted study subjects to non-smokers. Our a priori hypothesis is that individuals with yellow tongue coating have the higher prevalence of diabetes mellitus. Thus, we examined the association between yellow tongue coating and the prevalence of type 2 diabetes in the general population.

## METHODS

### Study population

The present study was a cross-sectional study, conducted as part of the Toon Health Study, a prospective cohort study in Toon City, Ehime Prefecture, Japan. The Toon Health Study commenced in 2009 and aimed to characterize risk factors for cardiovascular disease for its prevention in a community setting.^24^^,^^25^ In the Toon Health Study, we recruited participants from approximately 22,000 residents in Toon city who were 30–79 years old using newspaper advertisements, posters, and invitations. In all, 2,032 men and women participated in the baseline survey, conducted from 2009 through 2012. The examination of yellow tongue coating was conducted for the participants in 2011, 2012, and 2014. A total of 1,302 participants (465 men and 837 women) aged 30–79 years were enrolled in the study. We excluded current smokers (*n* = 121), and those not taking the oral glucose tolerance test (OGTT) due to the treatment for diabetes mellitus or having a prior history of total gastrectomy (*n* = 64). Participants who did not undergo the tongue inspection (*n* = 4) and those who removed their own tongue coating habitually (*n* = 139) or displayed a discolored tongue coating due to eating and drinking coffee or tea before their health examination (*n* = 5) were also excluded. In total, 315 men and 654 women were eligible for the analyses. The Institutional Review Board of Ehime University Graduate School of Medicine and Ethics Committee of the Juntendo University approved the study protocol, and informed consent was obtained from each study participant.

### Assessment of yellow tongue coating

All participants were asked whether they usually brushed off their tongue coating (yes/no) and whether they drank coffee or tea before their health examination (yes/no). Participants were then instructed to poke their tongue out as much as possible so the dorsum of the tongue could be photographed by a digital single-lens reflex camera (D50; Nikon Corp., Tokyo, Japan) with an electronic ring flash (Mark-2; IHS Co., Ltd., Niigata City, Japan). All photo images were taken under the light of fluorescent lamps in the same room and imaging environment. Each photo image included color charts (CasMatch; BEAR Medic Corp., Tokyo, Japan) in the image field and was adjusted for color by Photoshop CS2 (Adobe Systems, San Jose, CA, USA). A nationally licensed acupuncturist photographed and adjusted each tongue image and used these images to view the density of yellowness of tongue coating. Tongue coating was classified into three categories: white (normal), light yellow, and yellow tongue coating (eAppendix 1). The assessment of yellow tongue coating was conducted with blinding to the laboratory findings.

Furthermore, the yellowness of tongue coatings was quantified using the Commission Internationale de l’Eclairage L^*^a^*^b^*^ (CIELAB) and L^*^C^*^h° (CIELCH) color models. Recently, these indices are used for color segmentation in tongue diagnosis from digital images.^26^^–^^29^ In the CIELAB color model, L^*^ represents lightness (0: black to 100: white) and a^*^ (+a^*^: redness to −a^*^: greenness) and b^*^ (+b^*^: yellowness to −b^*^: blueness) represent color-opponent dimensions.^28^^,^^30^ Additionally, CIELAB provided the basis for determining hue-angle values, which were calculated with reference to the *a*-axis.^29^ The hue-angle value is used as an index of the CIELCH color model, in which L^*^ represents lightness (0: black to 100: white), C^*^ represents chroma (0: completely unsaturated to 100: very high chroma) and *h*° represents hue in degrees (or angles), ranging from 0° (red) through 90° (yellow), 180° (green), 270° (blue), and back to 0°. This study adopted CIE b^*^ and hue-angle values to quantify the yellowness of tongue coatings. Based on the RGB color model, the CIELAB was determined by taking measurements from all images of mean red, green, and blue values of 100 pixels in a square situated from the center to the back of the tongue dorsum, where tongue coating is most likely to be distributed.^14^ The formula for calculating CIELAB and hue-angle values from the RGB color model is shown in eAppendix 2.

### Measurement of oral glucose tolerance test and glycosylated hemoglobin A_1_c

Serum glucose levels during fasting and after the OGTT, as well as glycosylated hemoglobin A_1_c (HbA_1_c), were measured. All participants were required to fast for at least 10 hours before the OGTT. The reference starch solution (Toleran-G75; Ajinomoto Pharma Co., Ltd., Tokyo, Japan), containing 75 g of glucose, was orally administered in a fasting state. Venous blood samples were obtained at baseline, 1 hour, and 2 hours after the ingestion of 75 g glucose. In this study, the participants were classified using the recommendation of the American Diabetes Association in 2003.^31^ Diabetes mellitus was defined as either a fasting serum glucose level of ≥7.0 mmol/L or a 2-hour post-load glucose level of ≥11.1 mmol/L. Prediabetes was defined as a fasting serum glucose level of 5.6 mmol/L to 6.9 mmol/L, or 2-hour post-load glucose level of 7.8 to 11.0 mmol/L. HbA_1_c was measured using the latex immuno-agglutination method (Determiner hemoglobin A1c; Kyowa Medex Co., Ltd., Tokyo, Japan), and presented as the National Glycohemoglobin Standardization Program value.

### Assessment of covariates

Height and weight were measured in stocking feet with light clothing. Body mass index (BMI) was calculated as weight (kg) divided by the square of height (m^2^). Trained dietitians interviewed the participants to assess drinking status (never, former or current drinker), smoking status (never, former or current smoker) and physical activity. As a measure of physical activity, the Metabolic Equivalents of Task metric was assessed using the Japan Arteriosclerosis Longitudinal Study Physical Activity Questionnaire.^32^ Research physicians also asked participants whether they were using antidiabetic medication.

### Statistical analysis

Fasting and 1- and 2-hour post-load glucose levels were skewed; therefore, the variables were transformed using natural logarithms. Following statistical analysis, logarithmic values were transformed back to their original unit. Age- and sex-adjusted mean values for BMI, physical activity, fasting, 1- and 2-hour post-load glucose, and HbA_1_c levels, as well as the proportions of current drinkers and subjects with diabetes mellitus and prediabetes, were calculated using the analysis of covariance among yellow tongue coating categories and compared using post-hoc Dunnett’s test, with white tongue coating as the reference group. Multivariable logistic regression analyses were used to calculate the odds ratios (ORs) and 95% confidence intervals (CIs) for diabetes mellitus and prediabetes according to yellow tongue coating categories. Confounding factors included in the multivariable model were sex, age (years), BMI (kg/m^2^), drinking status (never, former, or current drinker), and physical activity (MET-hrs/day). The interactions of yellow tongue coating with sex in relation to diabetes mellitus and prediabetes were tested using cross-product terms of these variables in a logistic regression model. The sensitivity analysis for the association of yellow tongue coating with the prevalence of diabetes mellitus and prediabetes was performed using HbA_1_c levels according to the previous study, which showed optimal HbA_1_c cutoff of ≥6.0% as diabetes mellitus (sensitivity 83.7%, specificity 87.6%) and 5.7% to 6.0% as prediabetes (sensitivity 60.6%, specificity 72.1%) for the Japanese population.^33^ All statistical analyses were performed using SAS version 9.4 software (SAS Institute Inc., Cary, NC, USA). All *P*-values were two-tailed, and values <0.05 were considered statistically significant.

## RESULTS

Table 1 shows the characteristics of participants according to tongue coating categories. The proportion of subjects was 48.3% for white, 31.0% for light yellow, and 20.7% for yellow tongue coating. Compared to the white tongue coating, participants with the yellow tongue coating were older and more likely to be men. Mean CIE b^*^ and hue-angle values were significantly higher in the light yellow and yellow than the white tongue coating. Age- and sex-adjusted proportions of diabetes mellitus for white, light yellow, and yellow tongue coating were 5.8%, 6.9% and 10.6%, and those of prediabetes were 29.3%, 31.6%, and 34.9%, respectively.

**Table 1.** Characteristics of participants according to tongue coating categories

	White	Light yellow	Yellow	*P* value
	(*n* = 468)	(*n* = 300)	(= 201)	
Mean (SD) age, years	56.3 (13.6)	58.4 (12.3)^*^	63.0 (11.0)^*^	<0.01
Men, %	25.2	32.7	49.3	<0.01
Body mass index, kg/m^2^	23.0	23.1	23.3	0.59
Current drinker, %	58.5	59.5	54.1	0.41
Physical activity, METs h/day	35.4	35.3	35.3	0.81
Fasting serum glucose, mmol/dL	5.17	5.19	5.18	0.87
1-h serum glucose, mmol/dL	8.12	8.20	8.27	0.75
2-h serum glucose, mmol/L	6.75	6.89	7.03	0.23
HbA_1_c, %	5.46	5.50	5.52	0.18
Diabetes mellitus or prediabetes, %	35.1	38.5	45.5^*^	0.03
Diabetes mellitus, %	5.8	6.9	10.6	0.09
Prediabetes, %	29.3	31.6	34.9	0.34
Mean CIE b^*^	9.2	11.1^*^	15.0^*^	<0.01
Mean CIE *h*°	24.3	32.8^*^	46.0^*^	<0.01

CIE, Commission Internationale de l’Eclairage; HbA_1_c, glycosylated hemoglobin A_1_c; MET, metabolic equivalent; SD, standard deviation.

Values are adjusted for age and sex by the analysis of covariance. Age, CIE b^*^, CIE *h*° and sex values are shown as crude means and proportions.

^*^P < 0.05 as compared with white tongue coating (Dunnett’s test).

Table 2 shows multivariable ORs and 95% CIs of diabetes mellitus and prediabetes according to tongue coating categories. The multivariable ORs of diabetes mellitus were 1.39 (95% CI, 0.72–2.67) for light yellow tongue coating and 2.23 (95% CI, 1.16–4.30) for yellow tongue coating compared with white tongue coating. The multivariable ORs of prediabetes were 1.13 (95% CI, 0.80–1.61) for light yellow tongue coating and 1.43 (95% CI, 0.96–2.12) for yellow tongue coating. We further tested the effect modification by sex, because sex differences were observed among tongue coating categories. However, the interactions of sex on these associations did not reach statistical significance (*P* for interaction = 0.59).

**Table 2.** Multivariable odds ratios (ORs) and 95% confidence intervals (CIs) for diabetes mellitus and prediabetes according to tongue coating categories

	Number of subjects	Number of cases	Model 1		Model 2	
			OR (95% CI)	*P* value	OR (95% CI)	*P* value
NGT vs diabetes mellitus						
White	348	23	1.00		1.00	
Light yellow	209	20	1.37 (0.72–2.62)	0.34	1.39 (0.72–2.67)	0.33
Yellow	123	24	2.24 (1.17–4.29)	0.02	2.23 (1.16–4.30)	0.02
NGT vs prediabetes						
White	445	120	1.00		1.00	
Light yellow	280	91	1.15 (0.82–1.63)	0.42	1.13 (0.80–1.61)	0.48
Yellow	177	78	1.48 (1.00–2.18)	0.05	1.43 (0.96–2.12)	0.08

NGT, normal glucose tolerance.

Model 1: adjusted for age and sex.

Model 2: adjusted further for body mass index, drinking status, and physical activity.

In addition, we conducted a sensitivity analysis to examine the association of the yellow tongue coating with diabetes mellitus and prediabetes as defined by HbA_1_c. The multivariable ORs for diabetes mellitus were 1.32 (95% CI, 0.69–2.50) for light yellow tongue coating, and 2.13 (95% CI, 1.13–3.99) for yellow tongue coating compared with white tongue coating. The multivariable ORs for prediabetes were 1.55 (95% CI, 1.03–2.34) for light yellow tongue coating and 0.98 (95% CI, 0.59–1.61) for yellow tongue coating.

## DISCUSSION

The present cross-sectional study showed that yellow tongue coating was associated with higher prevalence of diabetes mellitus and tended to be associated with that of prediabetes among Japanese non-smoking men and women, even after adjustments for potential confounding factors. To our knowledge, this is the first study to show these associations among the general population. Half of the subjects showed light yellow or yellow tongue coatings in the present study. The proportion of these tongue coatings was similar to that reported in a previous study.^14^

Several clinical case reports showed a positive association between yellow tongue coating and diabetes mellitus.^11^^,^^12^ Fifty-three percent of diabetic nephropathy patients in a traditional Chinese medicine hospital displayed thin-greasy-yellow tongue coating.^11^ According to a case report of a diabetic patient with pyogenic liver abscess treated using integrative medicine, yellow tongue coating changed to white coating when blood glucose levels were improved and stabilized.^12^ Our study provides robust epidemiological evidence on the association between yellow tongue coating and diabetes mellitus.

The age- and sex-adjusted proportion of diabetes mellitus among individuals with yellow tongue coating was only 10.6%, although we showed a significant association between yellow tongue coating and higher prevalence of diabetes mellitus. The other individuals with yellow tongue coating did not have diabetes mellitus, probably because their tongue coatings were caused by other factors independent of diabetes mellitus, such as poor oral hygiene, coffee consumption, periodontal disease, oral candidiasis, and antibiotic medications.^13^^–^^17^

We validated the assessment of yellow tongue coating using CIE b^*^ and hue-angle values. The subjective assessment of tongue manifestations is common in clinical practice and research in traditional East Asian medicine, but several studies reported that CIE b^*^ and hue-angle values were significantly higher in groups with than without yellow tongue coatings,^27^^–^^29^ as shown in the present study.

Yellow tongue coating was also associated with higher prevalence of diabetes mellitus diagnosed using the optimal HbA_1_c cutoffs for Japanese population.^33^ However, no significant association was observed between yellow tongue coating and the prevalence of prediabetes based on HbA_1_c levels, probably because HbA_1_c levels are inadequate to identify prediabetes in Japanese population: the HbA_1_c cutoff of 5.7% for prediabetes has a high false-negative rate (39.4%).^33^

Although the mechanisms underlying the association between yellow tongue coating and diabetes mellitus remain unclear, previous studies postulated the following mechanisms. First, according to a next-generation sequencing study that examined differences in bacterial features between white and yellow tongue coatings, periodontal disease-related bacteria were more frequently identified in yellow (20 operational taxonomic units) than white tongue coatings (3 operational taxonomic units).^16^ Periodontal disease is known as a complication of diabetes mellitus via an inflammatory response due to hyperglycemia,^34^^,^^35^ while systemic inflammation due to periodontal disease may adversely affect glycemic control.^34^ Previous cohort studies showed that periodontal disease was associated with risk of diabetes mellitus.^36^^–^^39^ Additionally, a meta-analysis showed a significant reduction in HbA_1_c of 0.4% as a result of the periodontal treatment.^40^ Therefore, yellow tongue coating may be a clinical sign of periodontal disease related to an increased risk for diabetes mellitus. Second, yellow tongue coating is one of the manifestations of oral candidiasis.^15^ Oral candidiasis is known as a systemic complication of diabetes mellitus because high salivary glucose levels in patients with hyperglycemia enhance Candida colonization.^41^^,^^42^ A cross-sectional study also reported that oral candidiasis was more prevalent, even among patient with prediabetes, than healthy controls.^43^ Therefore, yellow tongue coating may be a clinical sign of oral candidiasis as a complication of not only diabetes mellitus but also the early stage of hyperglycemia.

One strength of the present study was the large sample size, which was enough to detect the association of yellow tongue coating with diabetes mellitus and prediabetes. Another strength is that we performed the OGTT to more precisely quantify diabetes mellitus and prediabetes, and we found that a yellow tongue coating was associated with diabetes mellitus and prediabetes after adjusting for potential confounding factors. Additionally, we quantified objectively the yellowness of tongue coating using CIE b^*^ and hue-angle values, which resolved significant linear associations.

Several limitations of the present study require discussion. First, this study had a cross-sectional design, so we could not demonstrate causality between yellow tongue coating and diabetes. Second, there might be other potential confounding factors affecting the observed associations. For example, certain medications, such as some antibacterial and psychotropic agents, cause tongue-coating discoloration.^17^ We did not assess use of such medications in the present study, though we excluded participants who were current smokers, had a habit of tongue brushing, or showed tongue coating discoloration due to tea or coffee and adjusted for other potential confounding factors. Third, the study participants were recruited voluntarily in the community, and current smokers were excluded. Smoking is a strong predictor for type 2 diabetes, and one in five people with type 2 diabetes was a current smoker.^44^^–^^46^

In conclusion, yellow tongue coating was associated with the higher prevalence of diabetes mellitus and also tended to be associated with that of prediabetes among Japanese non-smoking men and women. Yellow tongue coating, one of the clinical sings in traditional East Asian medicine, may be useful for the assessment and possibly for the prediction of diabetes mellitus. Prospective cohort studies will be necessary to examine whether yellow tongue coating is a predictor for diabetes mellitus.

## APPENDIX A. SUPPLEMENTARY DATA

eAppendix 1. Examples of tongue coating categories

eAppendix 2. Calculation of CIELAB and hue angle

## References

[1] Whiting DR, Guariguata L, Weil C, Shaw J IDF diabetes atlas: global estimates of the prevalence of diabetes for 2011 and 2030. Diabetes Res Clin Pract. 2011;94:311–321. 10.1016/j.diabres.2011.10.029 22079683

[2] Pagán JA, Tanguma J Health care affordability and complementary and alternative medicine utilization by adults with diabetes. Diabetes Care. 2007;30:2030–2031. 10.2337/dc07-0433 17519426

[3] Chang HY, Wallis M, Tiralongo E Use of complementary and alternative medicine among people with type 2 diabetes in Taiwan: a cross-sectional survey. Evid Based Complement Alternat Med. 2011;2011:983792. 10.1155/2011/983792 20953402 PMC2952338

[4] Yeh GY, Eisenberg DM, Kaptchuk TJ, Phillips RS Systematic review of herbs and dietary supplements for glycemic control in diabetes. Diabetes Care. 2003;26:1277–1294. 10.2337/diacare.26.4.1277 12663610

[5] Xie W, Zhao Y, Zhang Y Traditional Chinese medicines in treatment of patients with type 2 diabetes mellitus. Evid Based Complement Alternat Med. 2011;2011:726723. 10.1155/2011/726723 21584252 PMC3092648

[6] Xie W, Du L Diabetes is an inflammatory disease: evidence from traditional Chinese medicines. Diabetes Obes Metab. 2011;13:289–301. 10.1111/j.1463-1326.2010.01336.x 21205111

[7] Abuaisha BB, Costanzi JB, Boulton AJ Acupuncture for the treatment of chronic painful peripheral diabetic neuropathy: a long-term study. Diabetes Res Clin Pract. 1998;39:115–121. 10.1016/S0168-8227(97)00123-X 9597381

[8] Ji L, Tong X, Wang H, ; Evidence-Based Medical Research of Xiaoke Pill Study Group Efficacy and safety of traditional Chinese medicine for diabetes: a double-blind, randomised, controlled trial. PLoS One. 2013;8(2):e56703. 10.1371/journal.pone.0056703 23460810 PMC3584095

[9] Kim TH, Choi TY, Shin BC, Lee MS Moxibustion for managing type 2 diabetes mellitus: a systematic review. Chin J Integr Med. 2011;17:575–579. 10.1007/s11655-011-0811-2 21826591

[10] Zhang H, Tan CG, Wang HZ, Xue SB, Wang MQ Study on the history of Traditional Chinese Medicine to treat diabetes. Eur J Integr Med. 2010;2:41–46. 10.1016/j.eujim.2010.02.004

[11] Su K, Zhu F, Guo L, Zhu Y, Li W, Xiong X Retrospective study on Professor Zhongying Zhou’s experience in Traditional Chinese Medicine treatment on diabetic nephropathy. J Tradit Chin Med. 2013;33(2):262–267. 10.1016/S0254-6272(13)60137-5 23789229

[12] Liao PY, Hsu PC, Chen JM, Chiang JY, Lo LC Diabetes with pyogenic liver abscess—A perspective on tongue assessment in traditional Chinese medicine. Complement Ther Med. 2014;22:341–348. 10.1016/j.ctim.2013.12.009 24731906

[13] Gurvits GE, Tan A Black hairy tongue syndrome. World J Gastroenterol. 2014;20:10845–10850. 10.3748/wjg.v20.i31.10845 25152586 PMC4138463

[14] Mantilla Gómez S, Danser MM, Sipos PM, Rowshani B, van der Velden U, van der Weijden GA Tongue coating and salivary bacterial counts in healthy/gingivitis subjects and periodontitis patients. J Clin Periodontol. 2001;28(10):970–978. 10.1034/j.1600-051x.2001.028010970.x 11686816

[15] Pieralisi N, de Souza Bonfim-Mendonça P, Negri M, Jarros IC, Svidzinski T Tongue coating frequency and its colonization by yeasts in chronic kidney disease patients. Eur J Clin Microbiol Infect Dis. 2016;35:1455–1462. 10.1007/s10096-016-2684-y 27250632

[16] Jiang B, Liang X, Chen Y, Integrating next-generation sequencing and traditional tongue diagnosis to determine tongue coating microbiome. Sci Rep. 2012;2:936. 10.1038/srep00936 23226834 PMC3515809

[17] Abdollahi M, Rahimi R, Radfar M Current opinion on drug-induced oral reactions: a comprehensive review. J Contemp Dent Pract. 2008;9:1–15. 18335114

[18] Bastos AS, Leite AR, Spin-Neto R, Nassar PO, Massucato EM, Orrico SR Diabetes mellitus and oral mucosa alterations: prevalence and risk factors. Diabetes Res Clin Pract. 2011;92:100–105. 10.1016/j.diabres.2011.01.011 21300417

[19] Saini R, Al-Maweri SA, Saini D, Ismail NM, Ismail AR Oral mucosal lesions in non oral habit diabetic patients and association of diabetes mellitus with oral precancerous lesions. Diabetes Res Clin Pract. 2010;89:320–326. 10.1016/j.diabres.2010.04.016 20488573

[20] Collin HL, Niskanen L, Uusitupa M, Oral symptoms and signs in elderly patients with type 2 diabetes mellitus. A focus on diabetic neuropathy. Oral Surg Oral Med Oral Pathol Oral Radiol Endod. 2000;90:299–305. 10.1067/moe.2000.107536 10982950

[21] Alkhatib MN, Holt RD, Bedi R Smoking and tooth discolouration: findings from a national cross-sectional study. BMC Public Health. 2005;5:27. 10.1186/1471-2458-5-27 15790389 PMC1079878

[22] Agnihotri R, Gaur S Implications of tobacco smoking on the oral health of older adults. Geriatr Gerontol Int. 2014;14:526–540. 10.1111/ggi.12285 24697929

[23] Van Tornout M, Dadamio J, Coucke W, Quirynen M Tongue coating: related factors. J Clin Periodontol. 2013;40:180–185. 10.1111/jcpe.12031 23278504

[24] Tanno S, Tanigawa T, Saito I, Sleep-related intermittent hypoxemia and glucose intolerance: a community-based study. Sleep Med. 2014;15:1212–1218. 10.1016/j.sleep.2014.05.027 25156748

[25] Saito I, Hitsumoto S, Maruyama K, Heart rate variability, insulin resistance and insulin sensitivity in Japanese adults: the Toon Health Study. J Epidemiol. 2015;25:583–591. 10.2188/jea.JE20140254 26277879 PMC4549610

[26] Zhang B, Wang X, You J, Zhang D Tongue color analysis for medical application. Evid Based Complement Alternat Med. 2013;2013:264742. 23737824 10.1155/2013/264742 PMC3659485

[27] Xin-yu Y, Rong L, Zhao-ping W, Yu-jie R, Ying Z Current situation and analysis of a classification study of tongue color based on colorimetry. J Beijing Univ Trad Chin Med. 2012;35:539–542 (in Chinese).

[28] Su W, Xu ZY, Wang ZQ, Xu JT Objectified study on tongue images of patients with lung cancer of different syndromes. Chin J Integr Med. 2011;17:272–276. 10.1007/s11655-011-0702-6 21509670

[29] Xu Y, Zeng CC, Cai XY, Guo RP, Nie G, Jin Y Chromaticity and optical spectrum colorimetry of the tongue color in different syndromes of primary hepatic carcinoma. Zhong Xi Yi Jie He Xue Bao. 2012;10:1263–1271 (in Chinese). 10.3736/jcim20121110 23158945

[30] Bosman HH, Petkov N, Jonkman MF Comparison of color representations for content-based image retrieval in dermatology. Skin Res Technol. 2010;16:109–113. 10.1111/j.1600-0846.2009.00405.x 20384889

[31] Genuth S, Alberti KG, Bennett P, ; Expert Committee on the Diagnosis and Classification of Diabetes Mellitus Follow-up report on the diagnosis of diabetes mellitus. Diabetes Care. 2003;26(11):3160–3167. 10.2337/diacare.26.11.3160 14578255

[32] Ishikawa-Takata K, Naito Y, Tanaka S, Ebine N, Tabata I Use of doubly labeled water to validate a physical activity questionnaire developed for the Japanese population. J Epidemiol. 2011;21:114–121. 10.2188/jea.JE20100079 21258166 PMC3899503

[33] Shimodaira M, Okaniwa S, Hanyu N, Nakayama T Optimal hemoglobin A1c levels for screening of diabetes and prediabetes in the Japanese population. J Diabetes Res. 2015;2015:932057. 10.1155/2015/932057 26114121 PMC4465763

[34] Preshaw PM, Alba AL, Herrera D, Periodontitis and diabetes: a two-way relationship. Diabetologia. 2012;55:21–31. 10.1007/s00125-011-2342-y 22057194 PMC3228943

[35] Chávarry NG, Vettore MV, Sansone C, Sheiham A The relationship between diabetes mellitus and destructive periodontal disease: a meta-analysis. Oral Health Prev Dent. 2009;7:107–127. 19583037

[36] Saito T, Shimazaki Y, Kiyohara Y, The severity of periodontal disease is associated with the development of glucose intolerance in non-diabetics: the Hisayama study. J Dent Res. 2004;83:485–490. 10.1177/154405910408300610 15153457

[37] Demmer RT, Jacobs DR Jr, Desvarieux M Periodontal disease and incident type 2 diabetes: results from the First National Health and Nutrition Examination Survey and its epidemiologic follow-up study. Diabetes Care. 2008;31:1373–1379. 10.2337/dc08-0026 18390797 PMC2453650

[38] Ide R, Hoshuyama T, Wilson D, Takahashi K, Higashi T Periodontal disease and incident diabetes: a seven-year study. J Dent Res. 2011;90:41–46. 10.1177/0022034510381902 21041549

[39] Morita I, Inagaki K, Nakamura F, Relationship between periodontal status and levels of glycated hemoglobin. J Dent Res. 2012;91:161–166. 10.1177/0022034511431583 22157098

[40] Simpson TC, Weldon JC, Worthington HV, Treatment of periodontal disease for glycaemic control in people with diabetes mellitus. Cochrane Database Syst Rev. 2015;(11):CD004714. 26545069 10.1002/14651858.CD004714.pub3 PMC6486035

[41] Soysa NS, Samaranayake LP, Ellepola AN Diabetes mellitus as a contributory factor in oral candidosis. Diabet Med. 2006;23:455–459. 10.1111/j.1464-5491.2005.01701.x 16681553

[42] Hammad MM, Darwazeh AM, Idrees MM The effect of glycemic control on Candida colonization of the tongue and the subgingival plaque in patients with type II diabetes and periodontitis. Oral Surg Oral Med Oral Pathol Oral Radiol. 2013;116(3):321–326. 10.1016/j.oooo.2013.05.013 23953417

[43] Javed F, Ahmed HB, Mehmood A, Saeed A, Al-Hezaimi K, Samaranayake LP Association between glycemic status and oral Candida carriage in patients with prediabetes. Oral Surg Oral Med Oral Pathol Oral Radiol. 2014;117:53–58. 10.1016/j.oooo.2013.08.018 24332327

[44] Will JC, Galuska DA, Ford ES, Mokdad A, Calle EE Cigarette smoking and diabetes mellitus: evidence of a positive association from a large prospective cohort study. Int J Epidemiol. 2001;30:540–546. 10.1093/ije/30.3.540 11416080

[45] Akter S, Okazaki H, Kuwahara K, ; Japan Epidemiology Collaboration on Occupational Health Study Group Smoking, smoking cessation, and the risk of type 2 diabetes among Japanese adults: Japan Epidemiology Collaboration on Occupational Health Study. PLoS One. 2015;10(8):e0137039. 10.1371/journal.pone.0137039 26305358 PMC4549317

[46] Schipf S, Schmidt CO, Alte D, Smoking prevalence in type 2 diabetes: results of the Study of Health in Pomerania (SHIP) and the German National Health Interview and Examination Survey (GNHIES). Diabet Med. 2009;26:791–797. 10.1111/j.1464-5491.2009.02784.x 19709149

