# Supplementary material for: Yellow Tongue Coating is Associated With Diabetes Mellitus Among Japanese Non-smoking Men and Women: The Toon Health Study
Source: J Epidemiol. 2018 Jun 5;28(6):287–91. doi: 10.2188/jea.JE20160169 (PMC5976872; doi:10.2188/jea.JE20160169)
Supplement: Supplementary file 1 [file je-28-287-s001.pdf]

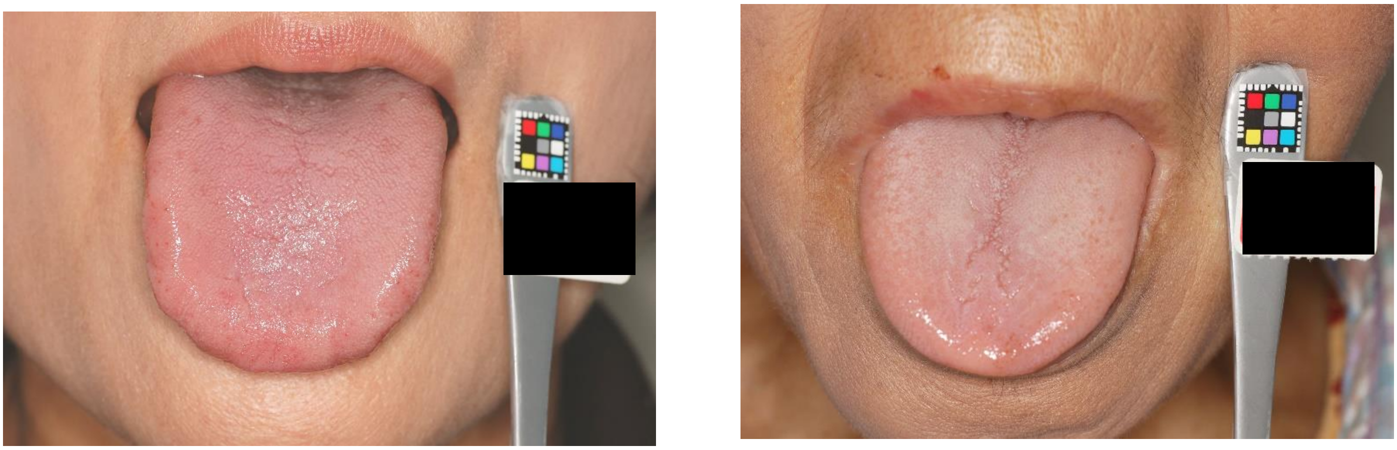

a) White tongue coating

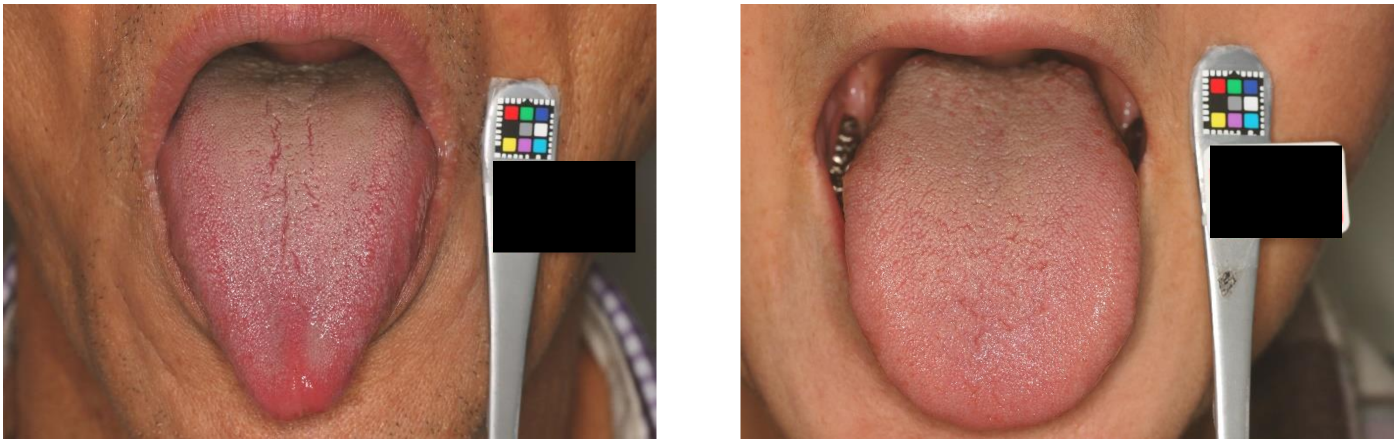

b) Light yellow tongue coating

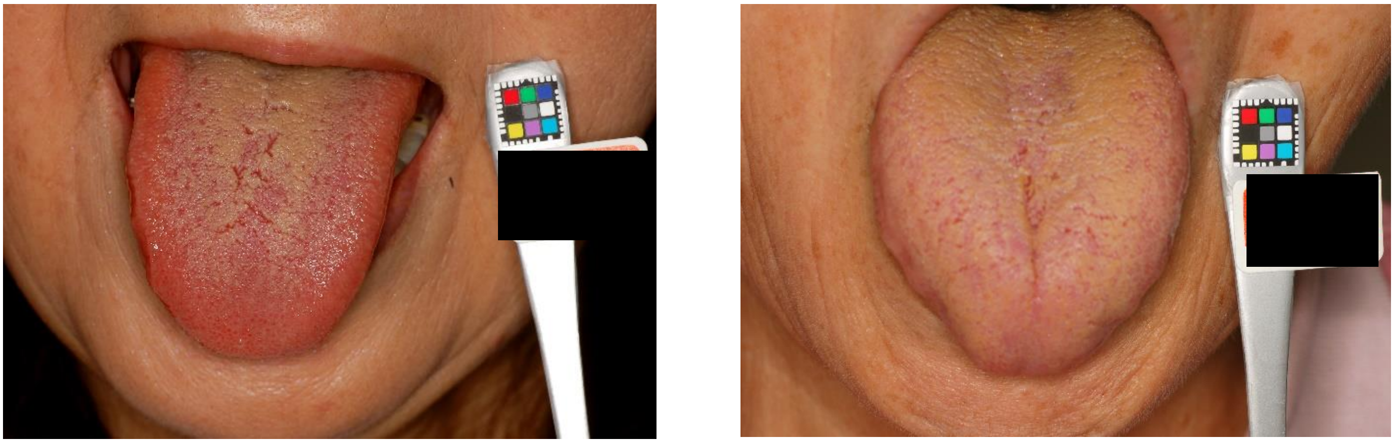

c) Yellow tongue coating

eAppendix 1. Examples of tongue coating categories

eAppendix 2. Calculation of CIELAB and hue angle

This study determined the CIELAB by measuring the mean RGB value of 100 pixels in a square situated from the center to the back of the tongue dorsum in all images. Each RGB value was converted (1) to CIELAB by first converting RGB to CIE*XYZ* using

$$\begin{bmatrix} X \\ Y \\ Z \end{bmatrix} = \begin{bmatrix} 0.4124 & 0.3576 & 0.1805 \\ 0.2126 & 0.7152 & 0.0722 \\ 0.0193 & 0.1192 & 0.9505 \end{bmatrix} \begin{bmatrix} \mathrm{R} \\ \mathrm{G} \\ \mathrm{B} \end{bmatrix}$$

followed by converting CIE*XYZ* to CIELAB via

$$L^* = 116 f\left(\frac{Y}{Y_0}\right) - 16,$$

$$a^* = 500\left[f\left(\frac{X}{X_0}\right) - f\left(\frac{Y}{Y_0}\right)\right], \quad (1)$$

$$b^* = 200\left[f\left(\frac{Y}{Y_0}\right) - f\left(\frac{Z}{Z_0}\right)\right],$$

$$\text{where f (x)} = \begin{cases} x^{1/3} & (x > 0.008856), \\ 7.787x + \frac{16}{116} & (x \le 0.008856). \end{cases}$$

In (1), X_0, Y_0, and Z_0 were the CIEXYZ tristimulus values of the reference white point. The hue angle was calculated using

$$\mathrm{h}° = \tan^{-1}\left(\frac{b^*}{a^*}\right),$$

and the unit was transformed from radians to degrees.

REFERENCE

1. Zhang B, Wang X, You J, Zhang D. Tongue color analysis for medical application. Evid Based Complement Alternat Med. 2013; 2013:264742.
